# Anaplastic Lymphoma Kinase in Cutaneous Malignancies

**DOI:** 10.3390/cancers9090123

**Published:** 2017-09-12

**Authors:** Severine Cao, Vinod E. Nambudiri

**Affiliations:** 1 Harvard Medical School, Boston, MA 02115, USA; severine_cao@hms.harvard.edu; 2 Department of Dermatology, Brigham and Women’s Hospital, Boston, MA 02115, USA

**Keywords:** anaplastic lymphoma kinase, cutaneous malignancy, anaplastic large cell lymphoma, crizotinib

## Abstract

Anaplastic lymphoma kinase (ALK) is a receptor tyrosine kinase that has been implicated in the pathogenesis of a variety of neoplasms. As suggested by its name, ALK was first described as part of a translocation product in cases of anaplastic large-cell lymphoma, with other genetic and cytogenetic ALK mutations subsequently coming to attention in the development of many other hematologic and solid organ malignancies. ALK has now been shown to play a role in the pathogenesis of several cutaneous malignancies, including secondary cutaneous systemic anaplastic large-cell lymphoma (ALCL) and primary cutaneous ALCL, melanoma, spitzoid tumors, epithelioid fibrous histiocytoma, Merkel cell carcinoma, and basal cell carcinoma. The characterization of ALK-positivity in these cutaneous malignancies presents exciting opportunities for utilizing ALK-targeted inhibitors in the treatment of these diseases.

## 1. Introduction

In the last two decades, new genetic and cytogenetic mutations in the tyrosine kinase, anaplastic lymphoma kinase (ALK), have been implicated in the pathogenesis of several neoplasms. This article will explore the role of ALK mutations in cutaneous malignancies, including its well-known association with systemic and primary cutaneous ALCL, as well as its emerging role in other cutaneous malignancies, such as melanoma, spitzoid melanocytic neoplasms, epithelioid fibrous histiocytomas, Merkel cell carcinoma, and basal cell carcinoma. The article will summarize the role of ALK in disease pathogenesis, as well as current efforts to develop ALK-targeted cancer therapies for these malignancies.

## 2. Discussion

### 2.1. Overview of ALK Biochemical Activity and Expression

Anaplastic lymphoma kinase (ALK) is a tyrosine kinase receptor that was first described in 1994 with the characterization of a frequently occurring translocation within a subset of patients with anaplastic large-cell lymphoma (ALCL) [1]. Since its initial description, translocations and other mutations of ALK have been implicated in the pathogenesis of a variety of malignancies, including lymphomas, neuroblastoma, non-small cell lung cancer, renal cell carcinoma, and colon carcinoma, pointing to its potential importance in the development of neoplastic processes [2].

ALK is a classic tyrosine kinase receptor, comprised of an extracellular binding domain, a transmembrane domain, and an intracellular tyrosine kinase domain with close homology to leukocyte tyrosine kinase [3,4]. The physiologic function of ALK remains poorly understood and is the subject of ongoing molecular investigations. Animal studies have characterized an expression pattern limited to specific regions of the central and peripheral nervous system, with mRNA and protein levels highest before birth [5,6,7,8]. Postnatal expression is relatively restricted to select cell types, including glial cells, endothelial cells, and pericytes [7]. Studies in cell lines and animal models have suggested a role for ALK in neuronal differentiation and mitogenesis [9,10,11,12], although ALK does not appear to be required for viability, given reports of only mildly abnormal behavioral phenotypes in *ALK*-knockout mice [13,14].

An endogenous ligand for ALK remains under investigation. Two small heparin-binding growth factors, Midkine [15] and Pleiotropin [16], have previously been reported as activating ligands for ALK, though results have not been confirmed in follow-up studies [9,17]. Since then, heparin [18] as well as the small proteins FAM150A and FAM150B [19,20,21], have been under study as possible candidates for ALK ligands. FAM150A and FAM150B are widely expressed, though FAM150B mRNA is most highly expressed in the adrenal gland [21], pointing to its potential role in the pathogenesis of ALK-positive neuroblastoma. It is possible that heparin and FAM150A and FAM150B act as co-ligands to fully activate the ALK receptor, much in the way that heparin and fibroblast growth factors act in concert to signal via fibroblast growth factor receptors [21]. Further investigation is required to elucidate the endogenous interactions between ALK and its ligands.

For unknown reasons, the ALK locus appears to be a hotspot for translocations, with 22 different translocation partners identified and implicated in the pathogenesis of several cancer types [2]. In each case identified thus far, the regulatory regions of the partner gene drive transcription of a fusion product, which then dimerizes through a dimerization domain on the partner protein, resulting in trans-autophosphorylation and constitutive activation of the ALK kinase domain. At the macroscopic level, the activation of ALK has been shown to have tumorigenic effects both in vitro and in vivo [22,23,24,25]. A more granular understanding of the intersecting signaling steps that result in oncogenic transformation has been complicated by the many fusion products under study. However, multiple pathways appear to be involved. In particular, ALK overexpression has been shown to activate the Ras–ERK pathway to promote cell proliferation [26,27], and the JAK3–STAT3 [28,29] and PI3K–Akt pathways [30,31] to increase cell survival. Modulation of Rho family GTPases may also lead to cytoskeletal rearrangements to induce cell migration and invasion [32]. Aside from translocations, other genetic alterations in the *ALK* gene that have been characterized in cancer pathogenesis include activating point mutations, amplifications of the ALK locus, alternative transcription, and small deletions [2]. Regardless of the particular derangement, the postulated central role of ALK in driving cancer progression raises the exciting possibility of using targeted therapies to treat ALK-positive cancer types.

### 2.2. ALK Expression in Cutaneous Lymphomas

ALK has long been known to play a role in the pathogenesis of the non-Hodgkin T-cell lymphoma, ALCL, for which the initial characterization of the translocation t(2;5)(p23;q35) provided the basis for its discovery. While not expressed in normal hematopoietic cells, ALK expression has been detected in 30–60% of cases of systemic ALCL [33,34,35], resulting in the subcategorization of ALCL into ALK-positive and ALK-negative subtypes. Cutaneous presentations of ALCL come in two forms: as a secondary cutaneous process in the context of underlying systemic disease, or as a primary cutaneous lymphoma with skin-limited manifestations. ALK has been found to be variably involved in the pathogenesis of these two cutaneous forms.

#### 2.2.1. Secondary Cutaneous ALCL

Systemic ALCL is a form of non-Hodgkin T-cell lymphoma defined by the characteristic proliferation of large, pleomorphic lymphoid blasts with strong CD30 expression known as “hallmark cells”. The disease primarily arises in nodal lymph tissue, though frequently presents with extranodal involvement. The skin is the most common site of extranodal disease, with cutaneous manifestations reported in 20–30% of systemic ALCL cases [36,37].

Patients typically present with systemic symptoms, such as fever, reflecting the release of cytokines by tumor cells [36]. When present, skin manifestations represent the infiltration of hallmark cells into the dermis, and often take the form of solitary or multiple pink-to-yellow papules or nodules that may ulcerate [36,38]. Interestingly, skin lesions of secondary cutaneous ALCL may present after minor cutaneous trauma, such as arthropod bites, raising the possibility that inflammation triggered by trauma may draw malignant T cells into the skin [39]. Cutaneous findings can be the first presenting symptom before the onset of systemic symptoms. Therefore, familiarity with disease presentation is critical for early appropriate diagnosis and management [40,41]. Skin manifestations of secondary cutaneous ALCL may also arise as paraneoplastic processes, with cases of acquired ichthyosis [42] and erythroderma [43] documented in the literature.

Since the characterization of ALK expression in 30–60% of systemic ALCL cases, it has become clear that ALK-positive ALCL presents as a distinct clinicopathologic entity compared to ALK-negative ALCL. ALK-positive ALCL is more commonly diagnosed in the first three decades of life and demonstrates a male predominance, whereas ALK-negative ALCL tends to manifest in the fifth to sixth decades of life with no gender predilection [35,37,38,44]. ALK-positive ALCL also presents with more advanced stage III/IV disease in up to 60% of cases [44,45], though its prognosis with multi-agent chemotherapy tends to fare better with 5-year survival rates ranging between 71–100% compared to 15–45% for ALK-negative ALCL [35,44,46,47,48,49]. Multivariate analyses have further demonstrated that the better prognosis holds, regardless of the younger age of diagnosis, with ALK expression independently related to an excellent prognosis in individuals with ALCL [38,40,41]. Studies of the frequency of extranodal involvement in ALK-positive versus ALK-negative ALCL have reported conflicting results, with some studies demonstrating more frequent extranodal involvement in ALK-positive ALCL [44], and others finding no difference, but with a predominance of cutaneous involvement in ALK-negative ALCL [37,50]. Ongoing research in this arena may help provide patients and their physicians with additional insight regarding the prognostic implications of ALK expression on the burden of cutaneous disease.

Among the 30–60% of systemic ALCL cases positive for ALK, 70–80% express the t(2;5)(p23;q35) translocation [36]. The translocation results in fusion of the *ALK* gene on chromosome 2 with the nucleophosmin (*NPM1*) gene on chromosome 5 [1]. Fusion between the two genes brings the regulation of ALK transcription under the strong *NPM* gene promoter, resulting in ectopic expression of a constitutively active NPM–ALK hybrid protein [26,51,52]. While the NPM–ALK translocation is by far the most common ALK translocation, an estimated 15–28% of ALK-positive ALCLs involve a fusion partner other than NPM [33,53,54]. Preliminary findings have suggested that variant translocations are associated with a better prognosis than the classical t(2;5) translocation [54]. Further study is required to compare the various ALK translocations, in terms of their cutaneous presentations, which has not been investigated to date, and has proven challenging given their relative infrequency.

Although epidemiologic data are incomplete regarding the predominance of cutaneous involvement in ALK-positive ALCL, in vitro experiments have outlined potential pathophysiologic mechanisms for ALK-mediated epidermotropism. ALK-positive lymphoma cells have been shown to release the pro-inflammatory cytokine high-mobility-group box-1 (HMGB-1), which is thought to promote the creation of a “premetastatic niche” within the skin [55]. HMGB-1 in turn promotes the secretion of IL-8 and MMP-9 by keratinocytes, which result in epidermal inflammation and the invasiveness of ALK+ lymphoma cells, respectively [56]. These findings offer hope for additional insights into the characterization of processes leading to secondary cutaneous involvement and properties for targeted therapy to address skin involvement in cases of ALK-positive systemic ALCL.

#### 2.2.2. Primary Cutaneous ALCL

Primary cutaneous ALCL is a subtype of ALCL that is considered part of a spectrum of CD30+ cutaneous lymphoproliferative disorders. This group of skin diseases includes variants of lymphomatoid papulosis and CD30+ drug reactions. Primary cutaneous ALCL is characterized by its skin-restricted presentation, with absence of extra-cutaneous disease for at least 6 months after presentation. The disease usually arises in the sixth decade of life with a slight male predominance, with rare cases in children and young adults [57]. Lesions are similar in morphology with those seen in systemic ALCL, presenting with solitary or localized nodules or tumors that may ulcerate [38,58,59] (Table 1). Histologically, lesions also appear similar to findings of systemic ALCL, with dense dermal infiltrates of CD30 expressing hallmark cells. A subtype of neutrophil-rich primary cutaneous ALCL has been characterized that presents with purulent ulcerations and the formation of small abscesses [60]. Frequent sites of involvement include the head and extremities [59,61,62]. Rare involvement of the penis has been documented in two case reports, presenting with paraphimosis [63,64]. In 20% of cases, patients may present with multifocal disease with two or more lesions at multiple anatomic sites [59,62].

The prognosis in primary cutaneous ALCL is excellent. Spontaneous regression can be seen in up to 25% of cases, with a 5-year survival rate of >90% [59,61,62,65,66]. Factors portending a worse prognosis include involvement of the legs and a greater extent of disease [65]. Several cases of primary cutaneous ALCL with poor prognosis have also been described in association with a chronic history of atopic dermatitis, and indicates a need to monitor these patients closely [67].

In contrast to systemic ALCL, ALK expression is frequently negative in primary cutaneous ALCL [65,66,68,69,70]. Of the handful of cases featuring ALK expression in primary cutaneous ALCL, many demonstrated eventual progression to systemic involvement [71,72,73,74,75]. Thus, ALK positivity has become viewed as a marker of systemic disease that should prompt further work-up in ALK-positive individuals presenting initially with skin-limited disease.

However, more recent reports have documented cases of ALK-positive skin-limited ALCL in pediatric patients [76,77,78]. Such cases may have previously gone unnoticed, due to exclusion of pediatric cases from prior studies. In one case series, 5 of 6 patients were successfully treated by conservative measures with local excision and/or radiotherapy, raising the possibility that ALK positivity is again associated with a positive prognosis [76]. The discovery of this subpopulation of ALK-positive primary cutaneous ALCL among pediatric patients suggests that ALK may be a less valuable marker for systemic disease in children, and underscores the need to identify other more reliable markers to differentiate between cutaneous and systemic forms of disease.

### 2.3. Trial of Therapeutic ALK Inhibition for Cutaneous-Involved ALK Lymphoma

Traditionally, primary cutaneous ALCL has been managed by surgical excision and/or radiation therapy [61], with an anthracycline-based chemotherapy regimen most commonly employed for systemic ALCL [79]. However, the characterization of ALK as a driver mutation in cancer pathogenesis has led to the introduction of targeted ALK therapies in the treatment of ALK-positive ALCL. Given the restricted physiologic expression of ALK in only a few neuronal tissues, the inhibition of ALK for cancer therapy should theoretically have high specificity for tumor cells and result in few systemic toxicities.

Crizotinib is an oral small molecule inhibitor of ALK which works by competing for the ATP binding site of the ALK kinase domain [2,80]. Its efficacy and tolerability in cancer therapy was first demonstrated in ALK-positive non-small cell lung cancer (NSCLC) [81,82,83,84,85], ultimately leading to its approval by the Food and Drug Administration for use in NSCLC in 2013.

The study of crizotinib as a potential therapy for ALK-positive ALCL has lagged behind investigations in NSCLC, due to the relatively lower incidence of ALCL. Studies thus far have shown promising results in small cohorts of pediatric and adult populations, with a complete response and resolution of lymphoma-associated symptoms within days of initiating treatment [86,87,88,89]. Reported toxicities have been mild and have included transient visual flashes, mild elevation in liver-function tests, dizziness, peripheral edema, and neutropenia [87,88]. Clinical trials are currently underway to investigate the efficacy of crizotinib for advanced and refractory cases of ALK-positive ALCL in children and adults (Table 2).

While initial studies have been promising, use of crizotinib in treatment of NSCLC has already been complicated by the emergence of resistance [90]. Studies of patients with NSCLC who have relapsed on crizotinib have revealed several mutations in the ALK kinase domain that may interfere with drug binding [91,92,93,94]. A mutagenesis study of NPM–ALK identified several potential mutations that could similarly limit responses to treatment of ALK-positive ALCL with crizotinib [95]. Targeted therapies for ALK-positive lymphoma will thus require development of second generation small molecule inhibitors or combination therapies to prevent treatment failure. Second generation small molecule inhibitors, such as ceritinib, alectinib, and brigatinib, have been developed and studied in ALK-positive NSCLC [96], with clinical trials for these agents in ALK-positive ALCL currently undergoing recruitment (Table 2).

### 2.4. ALK in Other Cutaneous Malignancies

#### 2.4.1. Melanoma

In the last five years, the introduction of several novel targeted therapies, including BRAF inhibitors, MEK inhibitors, and immunotherapies, has dramatically improved patient outcomes in advanced melanoma. This success has prompted a search for other driver mutations that may be used as therapeutic targets. One potential candidate is ALK [97]. The possibility of ALK expression in melanoma was first characterized in acral melanomas among patients in Southern China [98]. ALK translocations were identified by fluorescent in situ hybridization in 4 out of 30 primary acral melanomas. The particular translocations involved were not specified. All four patients presented with ulcerations and were diagnosed at a mean age of 46, much below the mean age of 62 reported in a retrospective study of acral melanoma in Chinese patients [99]. While the sample size was small, the authors suggested that ALK translocations in acral melanomas may be associated with a poorer prognosis.

Since this study, several others have confirmed the presence of other types of ALK mutations in melanoma. ALK missense mutations have been reported in a systematic review of next-generation sequencing data from melanoma samples [100]. In this study ALK was identified as the most frequently mutated gene among “pan-negative” melanomas, or melanomas without recurrent mutations in five known driver genes *BRAF*, *NRAS*, *KIT*, *GNAQ*, and *GNA11*. Of the ALK mutations identified, four were predicted to have a medium to high impact on protein function by the Mutation Assessor, though whether or not they could result in increased ALK activity was not studied [100].

More recently, a novel isoform of ALK has been described in a subset of primary and metastatic melanomas [101,102]. The isoform, designated ALK^ATI^, is generated from a de novo alternative transcription site in ALK intron 19, and results in increased expression of a truncated protein consisting of only the intracellular tyrosine kinase domain. Experiments have demonstrated its capacity to stimulate growth-factor independent cell proliferation in vitro and tumorigenesis in vivo [101]. Given its identification in both primary and metastatic melanomas, it is possible that the ALK^ATI^ isoform participates in early oncogenesis and subsequently persists within metastases. The reported incidence of ALK^ATI^ among current studies is variable, and ranges from 2–11%, likely due to the different methodologies used for its detection [101,102,103]. Interestingly, in their examination of 16 ALK-positive melanomas, Busam et al. report that all were associated with the ALK^ATI^ isoform, and none harbored a translocation [102]. While limited again by sample size, they report that ALK-positive melanomas were primarily nodular, and composed predominantly of amelanotic epithelioid or mixed spindle and epithelioid cells [102].

Altogether, these data suggest an emerging role for ALK in the pathogenesis of melanoma. It is possible that different mutation types are involved in different subsets of melanoma, with ALK translocations involved in acral melanoma and the ALK^ATI^ isoform in nodular subtypes (Table 3). Further study will be required to characterize a more accurate frequency of ALK-positive melanomas, and whether they present with a distinct morphologic phenotype.

The potential for ALK as a therapeutic target for advanced melanoma has been addressed by Wiesner et al. who in their characterization of the ALK^ATI^ isoform, found that its activity could be inhibited by three ALK inhibitors (crizotinib, ceritinib, and TAE-684) when incubated with ALK^ATI^- expressing cell lines [101]. To date, no published studies have looked at the role of ALK inhibitors in the treatment of human melanoma. An ongoing trial is currently recruiting patients to evaluate the use of entrectinib, a potent inhibitor of the tyrosine kinases ALK, TRKA/B/C, and ROS1, for the treatment of solid tumors, including melanoma (ClinicalTrials.gov identifier NCT02568267).

#### 2.4.2. Spitzoid Tumors

Spitzoid tumors represent a group of melanocytic neoplasms that occur more commonly in young individuals and are characterized histologically by proliferations of large epithelioid and/or spindle-shaped melanocytes [115]. The group consists of a spectrum of melanocytic lesions that vary in their malignant potential, ranging from benign Spitz nevi to malignant spitzoid melanomas. Clinically, lesions appear as sharply circumscribed, dome-shaped, pink-to-red papules predominantly on the face or lower extremities [115].

The diagnosis of spitzoid tumors is hampered by a lack of objective criteria to distinguish between the different subtypes. Research efforts have turned to genomic studies to find unique genetic markers that may aid in diagnosis. Spitz tumors generally lack mutations in oncogenes classically associated with melanoma, such as NRAS, KIT, GNAQ, and GNA11, though distinct histopathologic forms have been found to harbor BRAF^V600E^ and HRAS mutations, and suggest a role for activating kinase pathways in tumorigenesis [104,105,116]. Recently, activating kinase fusions have been characterized in spitzoid tumors lacking mutations in BRAF or HRAS [104,105]. These kinase fusions, which include ROS1, NRK1, BRAF, RET, and ALK, appear in a mutually exclusive manner across the entire spectrum of spitzoid tumors, and were shown to have tumorigenic activity when transduced in cell lines [104]. Fusion partners to ALK included TPM3, NPM1, CAP-Gly domain-containing linker protein 1 (CLIP1), translocated promoter region (TPR), general transcription factor 3C polypeptide 2(GTF3C2), and dynactin (DCTN1), with the latter two representing new oncogenic fusions not previously reported to participate in cancer pathogenesis [104,105,106,107].

Interestingly, spitzoid tumors harboring ALK fusions exhibit distinct clinical and histologic features [105,106,107]. Tumors tend to appear as polypoid nodules presenting on the lower extremities. Histologically, a plexiform dermal growth of intersecting fascicles composed of fusiform melanocytes is seen [105,106,107]. These findings have led to suggestions that ALK-positive Spitz tumors represent a distinct subset of Spitz tumors [105]. However, given the involvement of ALK across the spectrum of all spitzoid tumors, the gene may have limited utility in helping to clarify the tumor’s malignant potential. Instead, further research identifying the specific characteristics of ALK-positive spitzoid lesions, and their distinct characteristics, may help elucidate distinction of spitzoid lesions suspicious for melanoma or other malignant neoplasms.

The potential use of ALK inhibitors in the treatment of spitzoid tumors has only been tested in vitro so far. Wiesner et al. added crizotinib to Melan-a cells expressing the DCTN1–ALK fusion gene, and found that the drug inhibited phosphorylation of the fusion protein and of the downstream signaling proteins ERK and S6 [104]. To date, no studies have investigated the possibility of ALK inhibitors in the treatment of spitzoid tumors, though this remains an interesting area for possible future therapeutic exploration.

#### 2.4.3. Epithelioid Fibrous Histocytoma

Epithelioid fibrous histiocytoma (EFH) is a cutaneous mesenchymal neoplasm that commonly presents as a solitary symmetrical nodule usually on the extremities of young to middle-aged adults [117]. EFH was originally thought to represent a morphologic variant of benign fibrous histiocytoma with prominent epithelioid morphology. However, the recent characterization of ALK fusion products in EFH has prompted reconsideration of EFH as a separate entity [118].

The involvement of ALK in EFH pathogenesis was first documented with the report of two cases of cutaneous mesenchymal neoplasms found to stain for ALK by immunohistochemistry [108]. At the time, the two cases were diagnosed as atypical fibrous histiocytoma, but in retrospect, were more likely to be consistent with EFH, given the presence of positive staining for ALK and large epithelioid cells admixed in a background of histiocytes and spindle cells. Since then, two additional cases of EFH have been reported, featuring the translocations *VCL–ALK* and *SQSTM1–ALK* [109], with a subsequent series of 33 cases of EFH reporting diffuse cytoplasmic expression of ALK in 88% of cases [110]. These findings support the separation of EFH as an independent entity separate from benign FH, due not only to differences in histology, but also to distinct underlying biology. Given the data to date, ALK expression may prove to be a valuable marker in helping distinguish EFH from related mesenchymal neoplasms in cases of diagnostic uncertainty.

#### 2.4.4. Merkel Cell Carcinoma

Merkel cell carcinoma is a highly aggressive neuroendocrine skin tumor that typically presents as a rapidly growing, painless, flesh-colored or bluish-red intracutaneous nodule in older adults with light skin types [119]. The malignancy is thought to arise from a combination of factors, including infection with Merkel cell polyomavirus (MCV), ultraviolet radiation exposure, and immunosuppression [119]. In a study of 32 cases of Merkel cell carcinoma, immunohistochemistry revealed ALK reactivity in 30 cases (93.8%) [111].

RNA sequencing of 26 Merkel cell carcinomas has further identified ALK as the most frequently overexpressed gene among 50 cancer-related genes [112]. Interestingly, the authors found no evidence of fusion events, raising the possibility that ALK overexpression in Merkel cell carcinoma arises from genetic or epigenetic events. ALK overexpression did not vary between MCV-positive and MCV-negative Merkel cell carcinoma, implying that ALK contributes to the disease independent of MCV infection.

Merkel cell carcinoma is well-known for its bleak prognosis with a high incidence of recurrence. The possible role for of ALK in tumorigenesis presents a valuable opportunity to investigate the use of targeted therapies in its treatment—as either a primary or adjunctive strategy in control of Merkel cell carcinoma—which has, as-of-yet, not been addressed in the literature.

#### 2.4.5. Basal Cell Carcinoma

The pathogenesis of basal cell carcinoma (BCC) is known to involve the sonic hedgehog pathway (SHH) [120]. Vismodegib, an FDA-inhibitor against the downstream transcription factor smoothened, was developed to target this pathway. However, vismodegib has been associated with limited efficacy, prompting the search for other potential therapeutic targets [121,122]. Microarray analyses have demonstrated increased expression of ALK in BCC keratinocytes relative to normal epidermal keratinocytes [113,114], although the specific mutation driving overexpression has not yet been elucidated.

The potential use of crizotinib in the treatment of BCC has been evaluated in vitro, with crizotinib reducing keratinocyte proliferation in part by suppressing the expression of signaling molecules downstream in the SHH pathway, GLI1 and CCND2 [114]. These results suggest that ALK activates downstream signaling molecules in parallel with the conventional SHH pathway, and opens the possibility of using ALK inhibitors, perhaps in combination with vismodegib, in the treatment of BCC. Further exploration of this pathway as a treatment target for the most common form of skin cancer is underway.

## 3. Conclusions

Since its discovery in 1994, the tyrosine kinase ALK has emerged as a key player in the pathogenesis of several malignancies, many of which present with cutaneous manifestations. In synthesizing the literature on ALK-associated cutaneous malignancies, this article aids in the early diagnosis and appropriate management of ALK-positive malignancies presenting with cutaneous findings. Further study will be required to fully characterize the clinical and prognostic implications of ALK positivity in cutaneous disease.

The characterization of ALK as a driver mutation has offered the exciting possibility of treating malignancies with ALK-targeted small molecule inhibitors. This has only just begun to be studied in the realm of cutaneous malignancies, with clinical trials currently ongoing for ALK-positive systemic ALCL and melanoma. As cancer treatment moves from “one-size-fits-all” cytotoxic therapies to more tailored approaches based on specific molecular alterations, tyrosine kinases like ALK will be expected to take center stage as therapeutic targets. It will be exciting to see the continued characterization of ALK in the pathogenesis of neoplastic processes and the development of novel strategies to inhibit its tumorigenic effects.

## Figures and Tables

**Table 1:** Similarities and differences between secondary cutaneous systemic anaplastic large-cell lymphoma (ALCL) and primary cutaneous ALCL.

	Secondary Cutaneous Systemic ALCL	Primary Cutaneous ALCL
*Role of ALK*	- ALK-positive in 30–60% of cases	- Frequently negative - ALK-positive in unknown proportion of pediatric cases
*Clinical Characteristics*	- Solitary or multiple papules or nodules that may ulcerate - Systemic symptoms present	- Solitary or multiple papules or nodules that may ulcerate
*Histology on Skin Biopsy*	- Dermal infiltrates comprising “hallmark cells”	- Dermal infiltrates comprising “hallmark cells”
*Age of Diagnosis*	- Typically dependent on ALK status: ALK-positive- first 3 decades of life ALK-negative- 5th–6th decade of life	- Typically 6th decade of life
*Gender Predominance*	- Dependent on ALK status: ALK-positive—Male ALK-negative—None	- Male
*Treatment*	- Antracycline-based chemotherapy regimen, e.g., CHOP (cyclophosphamide, doxorubicin, vincristine, prednisone)	- Surgical excision and/or radiation therapy
*Prognosis*	- Dependent on ALK status ALK-positive 5-year survival 71–100% ALK-negative 5-year survival 15–45%	- 5-year survival >90%

ALK: Anaplastic lymphoma kinase.

**Table 2:** Ongoing clinical trials investigating use of targeted therapies in ALK-positive cutaneous malignancies.

ClinicalTrials.gov Identifier	Phase	Treatment	Cutaneous Malignancy under Study	Population
NCT02419287	Phase 2	Crizotinib	Recurrent or refractory ALK-positive ALCL	18 years and older
NCT00939770	Phase 1 and 2	Crizotinib	Recurrent or refractory ALK-positive ALCL	1 year to 21 years
NCT01524926	Phase 2	Crizotinib	Locally advanced and/or metastatic ALK-positive ALCL	1 year and older
NCT01449461	Phase 1 and 2	Brigatinib	ALK-positive ALCL	18 years and older
NCT01742286	Phase 1	Ceritinib	ALK-positive ALCL	12 months to 17 year
NCT02568267	Phase 2	Entrectinib	ALK-positive ALCL and melanoma	18 years and older

**Table 3:** ALK in other cutaneous malignancies.

Cutaneous Malignancy	ALK Mutation	Clinical Presentation	Specific Histology	References
*Melanoma*	Translocation	Acral melanoma presenting with ulcerations	N/A	[98]
	Alternative splicing ALK^ATI^ isoform	Nodular melanoma	Malignant amelanotic epithelioid cells or mixed spindle and epithelioid cells	[101,102,103]
*Spitzoid tumors*	Translocation with partners *TPM3*, *NPM1*, *CLIP1*, *TPR*, *GTF3C2*, *DCTN1*	Clinically amelanotic or vascular appearing polypoid nodules on the lower extremities	Melanocytes arranged in a fascicular growth pattern oriented in a radial pattern, mostly amelanotic	[104,105,106,107]
*Epithelioid Fibrous Histocytoma*	Translocations with parterns *VCL* and *SQSTM1*	Solitary symmetric nodule on the extremities	Storiform pattern of spindle cells and histiocytes with prominent population of epithelioid cells	[108,109,110]
*Merkel Cell Carcinoma*	Unknown	Flesh-colored or bluish-red nodule	N/A	[111,112]
*Basal Cell Carcinoma*	Unknown	Pearly nodule with small telangiectasias and a rolled border in sun exposed areas	N/A	[113,114]
